# Effect of learning on slow gamma propagation between hippocampus and cortex in the wild-type and AD mice

**DOI:** 10.1038/s41598-022-26754-2

**Published:** 2022-12-26

**Authors:** Katarzyna J. Blinowska, Maciej Kamiński, Nathalie Macrez, Aline Marighetto, Pierre Meyrand, Tiaza Bem

**Affiliations:** 1 Nałęcz Institute of Biocybernetics and Biomedical Engineering, Polish Academy of Sciences, Warsaw, Poland; 2 Department of Biomedical Physics, Faculty of Physics, University of Warsaw, Warsaw, Poland; 3 University Bordeaux, INSERM, Neurocentre Magendie, U1215, 33000 Bordeaux, France

**Keywords:** Neurophysiology, Network models

## Abstract

Slow gamma oscillations (20–50 Hz) have been suggested to coordinate information transfer between brain structures involved in memory formation. Whereas the involvement of slow gamma in memory processing was studied by means of correlation between the gamma power and the occurrence of a given event (sharp wave ripples (SWRs), cortical transients), our approach consists of the analysis of the transmission of slow gamma itself. We use the method based on Granger causality principle—direct Directed Transfer Function, which allows to determine directed propagation of brain activity, including bidirectional flows. Four cortical sites along with CA1 ipsi- and contralateral were recorded in behaving wild-type and APP/PS1 mice before and after learning session of a spatial memory task. During slow wave sleep propagation of slow gamma was bidirectional, forming multiple loops of interaction which involved both CA1 and some of cortical sites. In episodes coincident with SWRs the number and strength of connectivity pathways increased in both groups compared to episodes without SWRs. The effect of learning was expressed only in APP/PS1 mice and consisted in strengthening of the slow gamma transmission from hippocampus to cortex as well as between both CA1 which may serve more efficient transmission of information from impaired CA1.

## Introduction

Cortico-hippocampal interaction during sleep is a neural basis for memory consolidation^1^ and a large quantity of work has been devoted to describe different modes of such interaction^2–7^. Memory replay is initiated during Sharp Wave Ripples (SWRs), high frequency oscillations (100–250 Hz) lasting around 40–60 ms that are generated in the hippocampus with a rate around 0.5 Hz. During SWRs spiking patterns generated by hippocampal cells during previous experiences are re-activated in a compressed time scale and each time this reactivation occurs, intercortical connections between the disparate, active cortical networks are gradually strengthened. This hypothesis was reinforced by the evidence that stimulation-produced suppression of SWRs impairs subsequent spatial memory^8–10^.

SWRs are coordinated in rodents^3^ and human^11^ with oscillating Up and Down states, wide spread activation and suppression of prefrontal cortex (PFC) activity, one of a major sites of hippocampal output. Importantly, during SWRs, cortical patterns of awake experiences are reactivated and accuracy of replay is maximal at the transition from Down to Up states^12^. Moreover, it was observed that post-SWR activation of anterior cingulate cortex (ACC) neurons correlated positively with ripple amplitude and the same neurons were excited upon electrical stimulation of the CA1, suggesting possible functional pathway^13^. Recent findings indicate also an engagement of retrosplenial cortex (RSC) as a bridge in the hippocampal–cortical interaction. Indeed, the existence of events analogues to hippocampal ripple in RSC and their coupling to hippocampal SWRs in a brain-dependent way was demonstrated^14^.

Interestingly, several findings point out that a slow gamma oscillation (20–50 Hz) may play important role in the coordination of information transfer between the hippocampus and cortex, Indeed, it was shown that increased synchrony of slow gamma across CA1 and CA3 regions observed during SWRs supports coordinated replay across hippocampal networks and reinsures the fidelity of recapitulated experiences^15^. In a more recent study, the power of slow gamma was found to be strongly correlated with Up and Down States of RSC, the activity of which predicted hippocampal gamma and followed SWRs^7^. Therefore, in the present study we focus on the analysis of the cortico-hippocampal interaction mediated by slow gamma.

Whereas previously the involvement of gamma oscillations in coordination of activity between hippocampus and cortex was studied by means of the correlation between gamma power and the occurrence of a given event (for example SWR’s occurrence or UP to Down transition in the cortex) (c.f.:^7,16^) our approach consists of the analysis of the transmission of the gamma oscillation itself. Indeed, if slow gamma plays an important role in cortico-hippocampal dialog it is interesting to follow the tract along which this oscillation is arriving, through direct or indirect projection, into a given population of neurons, where oscillation is initiated again and propagates elsewhere.

In our analysis we applied direct Directed Transfer Function (dDTF)^17^, a measure of transmission based on Granger causality principle, which allows estimating directed propagation of brain activity between different channels as a function of frequency. In our study brain activity is monitored in PFC, RSC and posterior and anterior cingulate cortices (PCC and ACC) as well as in 2 hippocampal sites: CA1 ipsilateral and contralateral. Whereas correlation coefficient and spectral coherence indicate the strength of interactions between signals, dDTF offers also information about the direction of interaction and is able to identify reciprocal connections, which is particularly important in the context of the transfer of information between given brain structures.

A large quantity of work has been devoted to understand the role of SWRs in memory formation in normal animals but only few papers address the question of the effect of Alzheimer disease (AD) pathology on the SWRs functional integrity and their involvement in a dialog between cortex and hippocampus^16,18–20^.

Alzheimer's disease is a neurodegenerative disease which is responsible for 50–70% of the cases of dementia. The absence of identified etiological causes and treatments, combined with epidemiological statistics, make AD an issue of major public health and justify the research effort focused on the disease. According to 2020 survey there were approximately 50 million people worldwide suffering from Alzheimer's disease. AD affects about 6% of people older than 65 years. Early symptoms are: difficulties in remembering recent events; they are followed by problems with language, disorientation, mood swings, loss of motivation, self-neglect. Finally physiological functions are lost, ultimately leading to death. AD imposes a large burden on family and society. There is no treatment, that stop or reverse AD progression, though some medication may temporarily alleviate its symptoms.

The origins of AD is still misunderstood although the literature concerning AD and its diagnosis in humans is very extensive e.g. position papers of expert panels^21,22^. Because of obvious limitations of the studies of human brain structures interactions the animal models of AD are useful. However, the complex pathogenesis and pathological mechanisms of AD, the differences between autosomal-dominant AD and sporadic AD, make that most of the animal models including APP/PS1 model can only simulate part of the pathological characteristics of AD. The APP/PS1 transgenic mice reproduce some of the neuropathological lesions of AD, in particular cerebral amyloidosis and amyloid angiopathy, as well as early synaptic deficit and learning and memory deficits^23,24^. APP/PS1 has also been proposed as an alternative tool for AD research based on intravital imaging study of the brain^25^.

At the level of neuronal network, the comparison of the methods of connectivity analysis for AD diagnosis was reported in^26^ where as the most effective method was indicated to be the full frequency DTF (ffDTF). We have recently shown that APP/PS1 mice (in the following we shall call them TG—transgenic) are able to learn a spatial reference memory task despite a major deficit in SWRs^27^ and that reconfiguration of cortical–hippocampal interaction may be an adaptive mechanism bypassing Aβ-plaques impact and allowing a less efficient though operational spatial memory consolidation in APP/PS1 mice model^20^.

In the present paper, using similar methodology, we analyze interaction patterns expressed during slow wave sleep (SWS) before and after one day learning of a spatial discrimination task in the wild type (WT) and TG animals. Herein, we studied the transmission of slow gamma between all six recorded structures during periods coincident with ripples detected in CA1, we will call them (SWS/SWR) and in the absence of ripples (SWS/no-SWR).

Our results show, counterintuitively, that after learning session alteration of patterns of slow gamma transmission between hippocampus and cortex expressed during SWRs is more important in TG than in WT mice.

## Results

**Figure 1:**
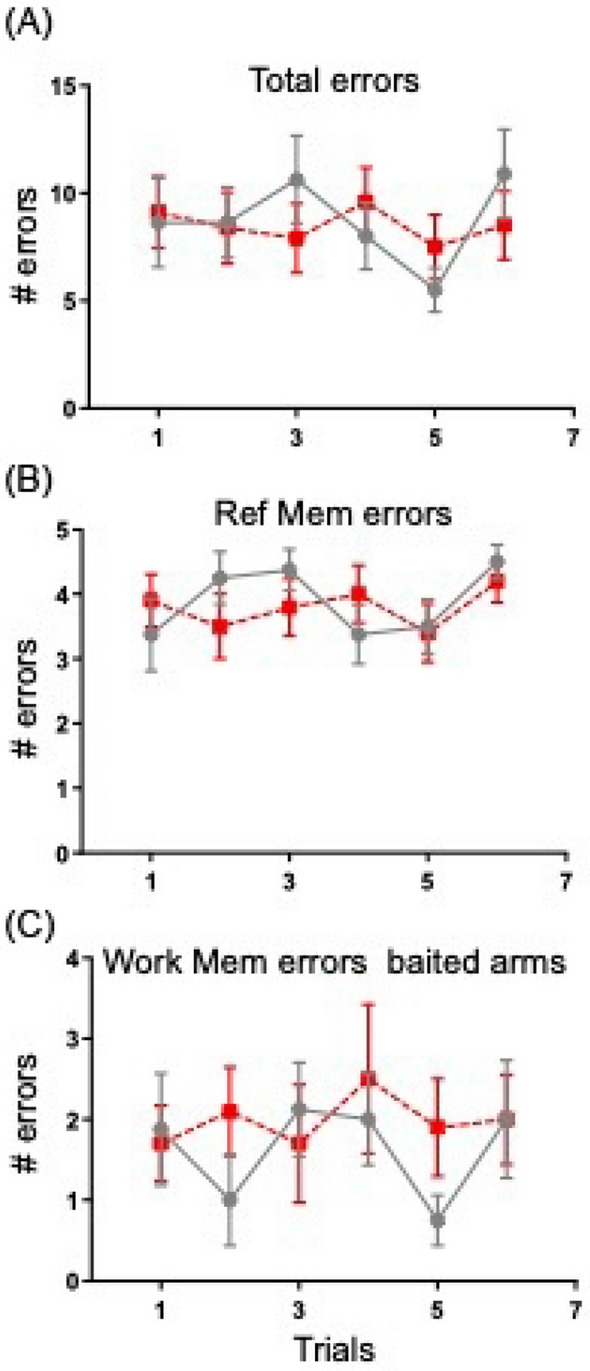
Number of errors made during one day of spatial discrimination task. Illustrated are: total errors (**A**), Reference Memory Errors (**B**) and Working Memory errors (**C**) made during 6 consecutive trials. Notice lack of difference in the performance between the WT (black curve) and TG (red curve) mice.

Performance of the spatial memory task was similar in both group of animal as illustrated in Fig. 1. There was no statistical difference at day 1 between WT and TG mice although TG already start to make more Working Memory errors in the last trials.

**Figure 2:**
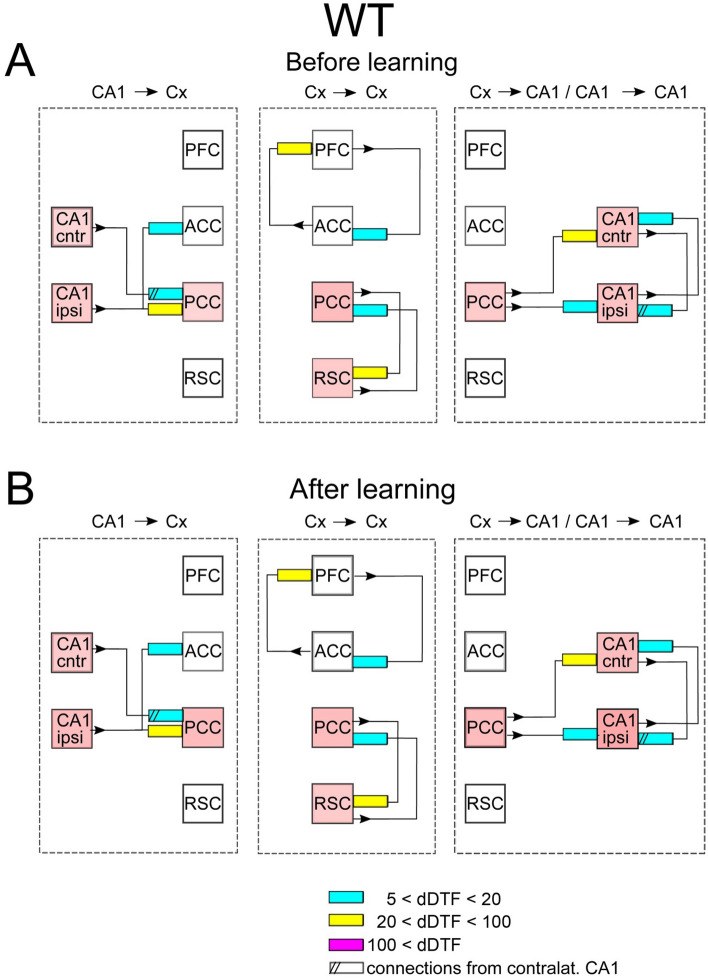
Scheme of interaction between brain structures in WT mice in the absence of SWRs. Shown are connections expressed before and after learning. The mean strength of the interactions within the group (in arbitrary units) is coded by colors of rectangles pointing out to the target structures; see the insert in the figure. The numbers in the insert correspond to dDTF values integrated over 20–50 Hz multiplied by 10^5^. Pink color marked structures involved in hippocampal–cortical–hippocampal loop of transmission of slow gamma signals, here realized by: CA1 → PCC → RSC → PCC → CA1 transmission. Notice the disconnection of signals transmitted in the PFC → ACC → PFC loop from the main loop of transmission.

**Figure 3:**
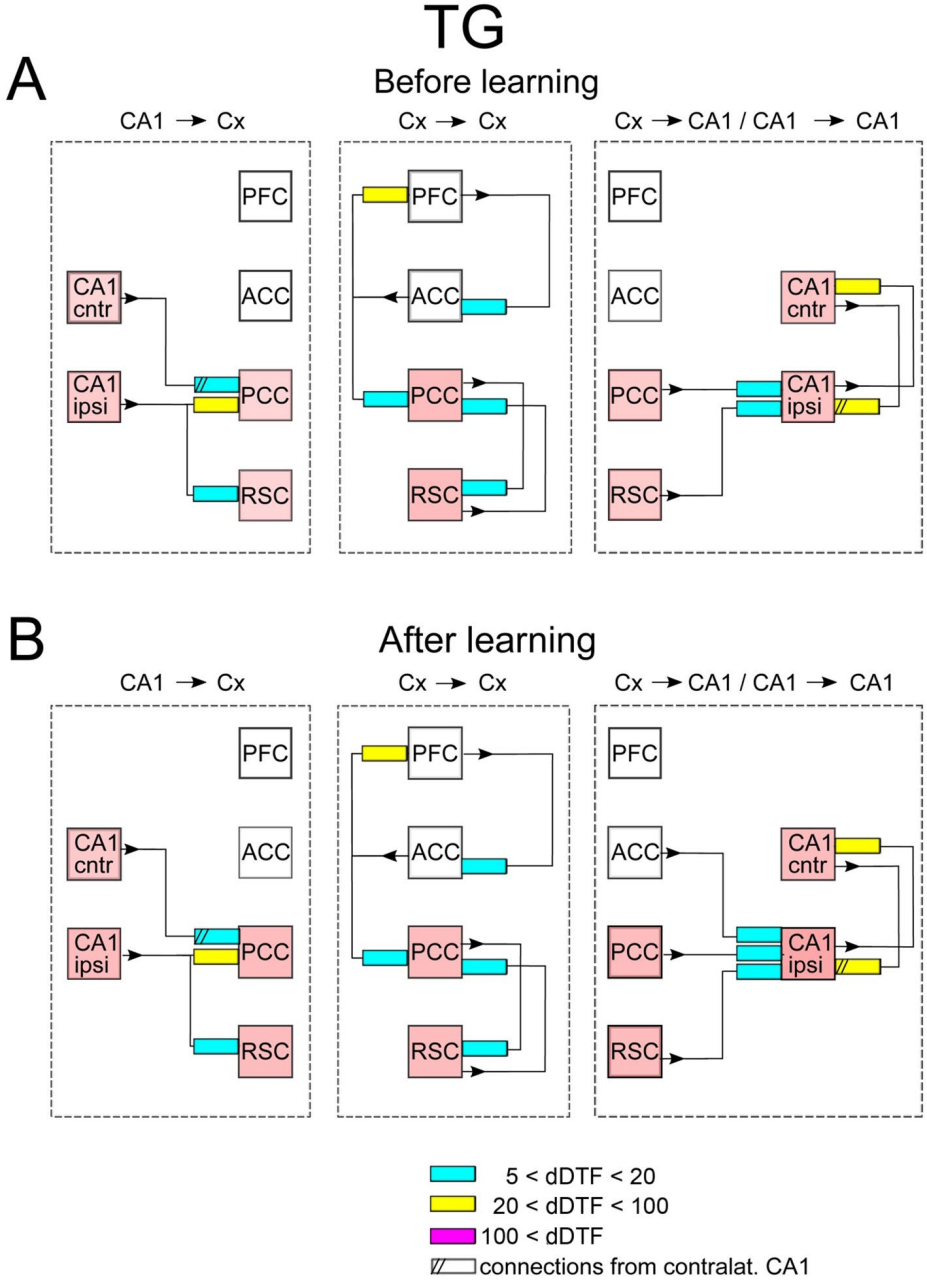
Scheme of interaction between brain structures in TG mice in the absence of SWRs. Shown are connections expressed before and after learning. Notice one way influence from PFC → ACC → PFC loop on the main loop of slow gamma transmission marked by pink squares. The organization of the picture as in Fig. 2.

**Figure 4:**
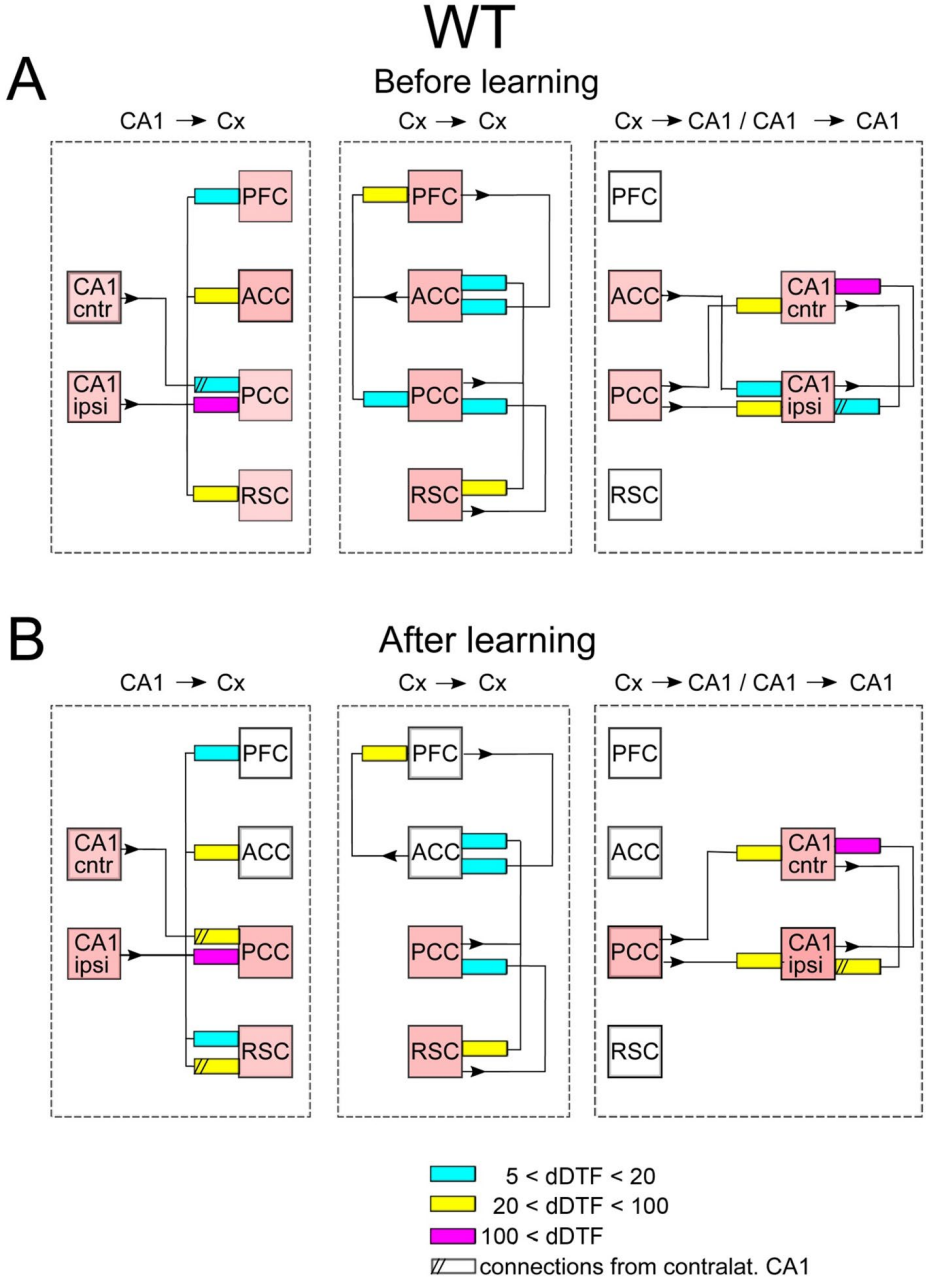
Scheme of coupling between brain structures in WT mice during SWRs. Before learning session slow gamma oscillations are synchronized among all cortices as indicated by pink squares. Notice that after learning the loop PFC → ACC → PFC becomes partly disconnected from the main loop of hippocampal–cortical–hippocampal transmission. The organization of the picture as in Fig. 2.

**Figure 5:**
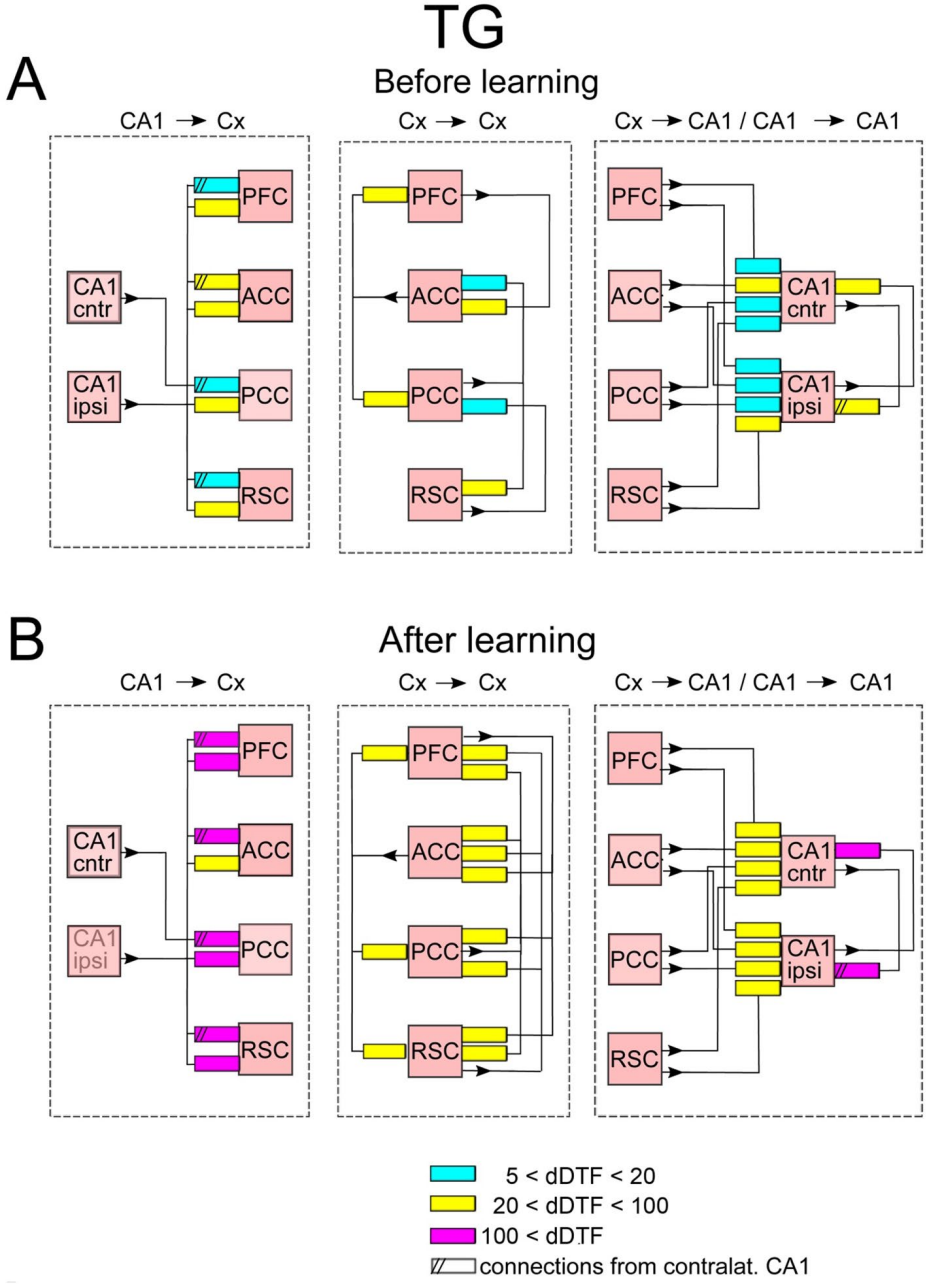
Scheme of coupling between brain structures in TG mice during SWRs, before learning and after learning. Notice extensive interaction between all structures which is even strengthen after learning session. The organization of the picture as in Fig. 2.

**Figure 6:**
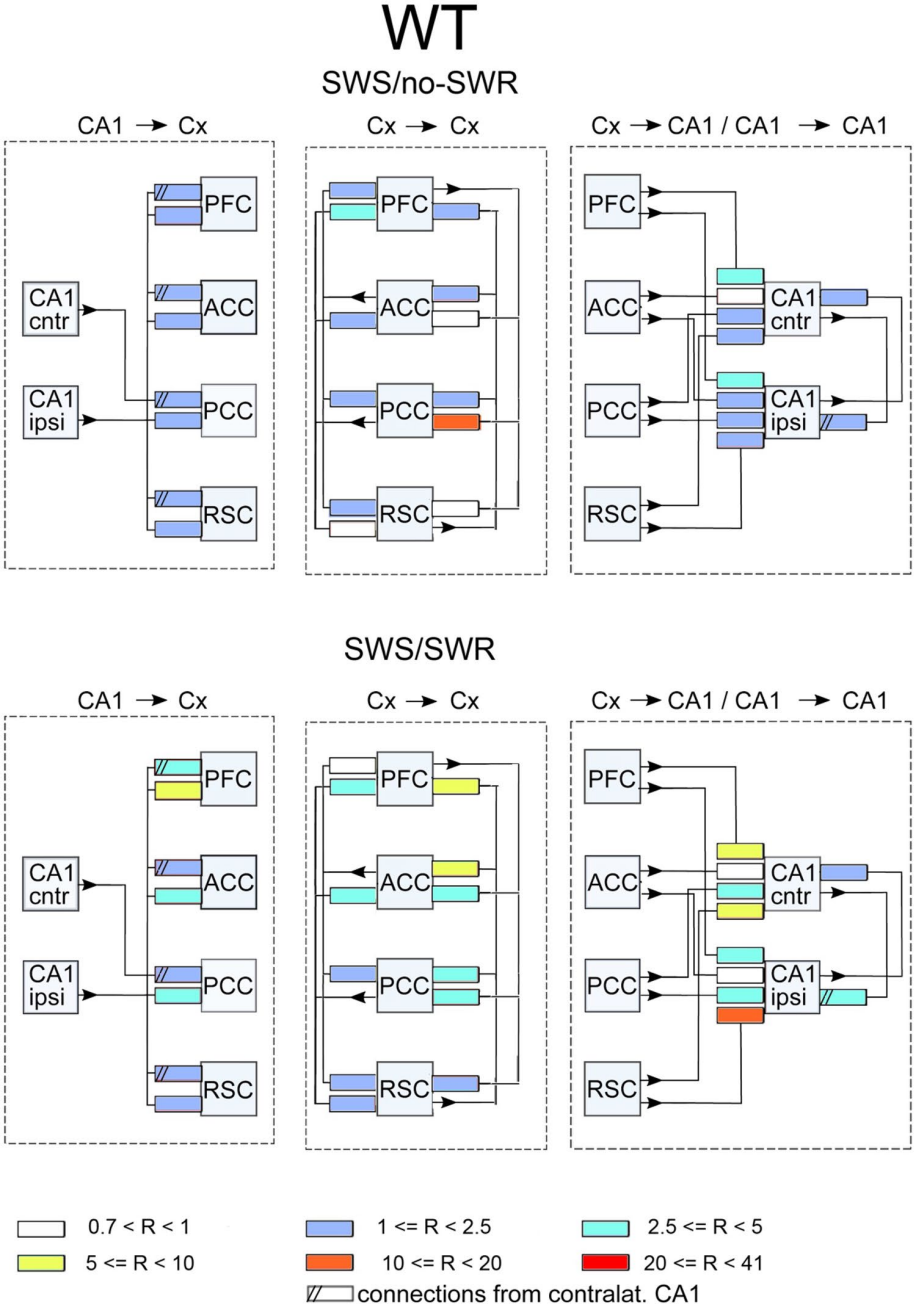
The ratios of coupling for WT mice after/before learning for SWS/no-SWR episodes (upper picture) and for SWS/SWR episodes (lower picture). Shown are (from the left to the right): coupling ratios from CA1 to cortex, between cortices, from cortices to CA1, and between two CA1. The color coding of values of the ratios at the bottom of the figure.

**Figure 7:**
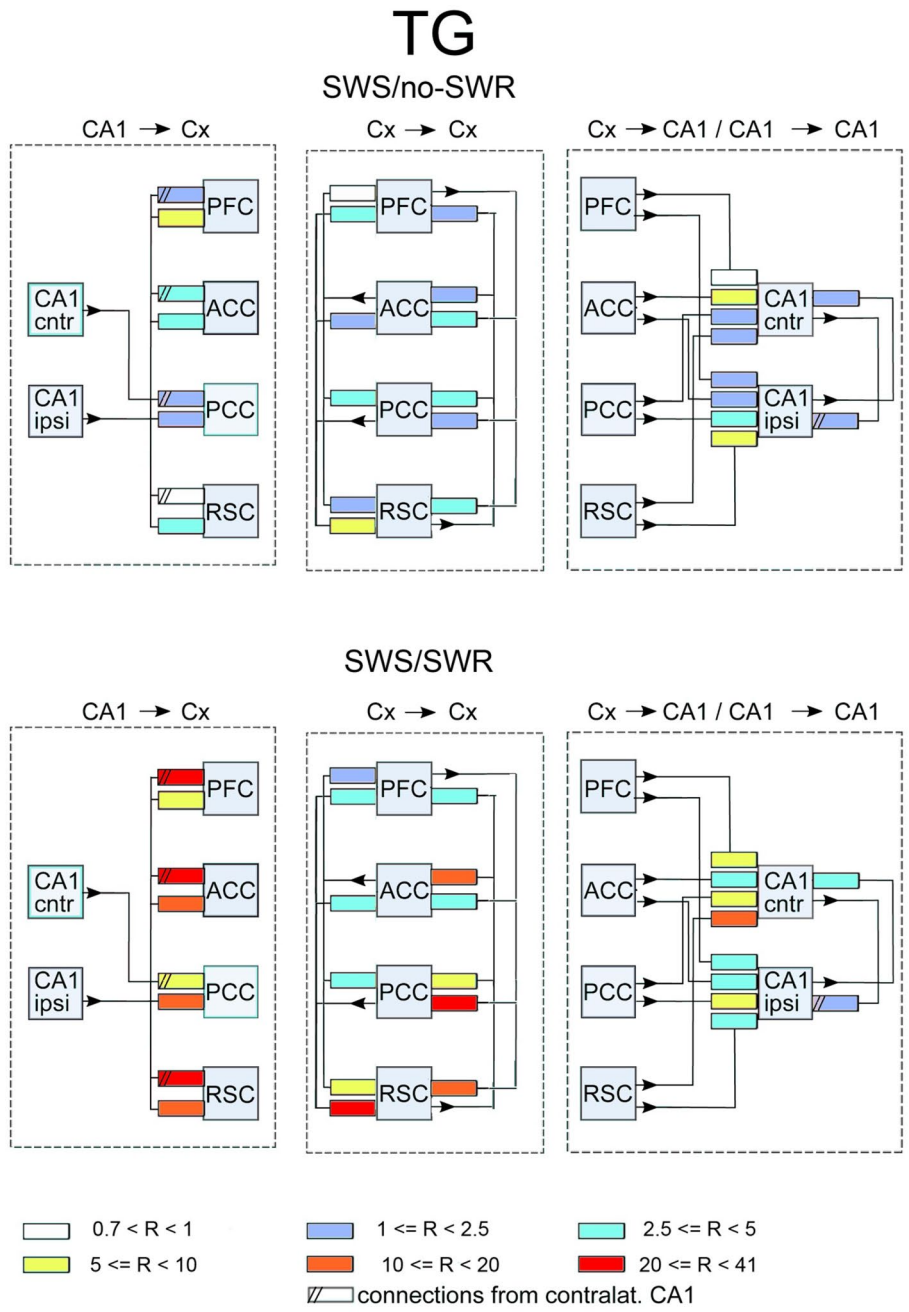
The ratios of coupling for GT mice after/before learning for SWS/no-SWR episodes (upper picture) and for SWS/SWR episodes (lower picture). Shown are (from the left to the right): coupling ratios from CA1 to cortex, between cortices, from cortices to CA1, and between two CA1. The color coding of values of the ratios at the bottom of the figure.

In each experimental condition, i.e. before or after learning, the transmission of slow gamma oscillations (20–50 Hz) between recorded structures was calculated in two ways: taking into account only time intervals of slow wave sleep without ripples (SWS/no-SWR) or considering only SWS episodes corresponding to SWRs’ occurrence in CA1 (SWS/SWR). Indeed, due to a particular role of SWRs in transmitting information from hippocampus to cortex in “replay” events we were particularly interested in comparison of the cortico-hippocampal interaction patterns expressed during and in the absence SWRs. It is worth noticing that an individual SWR lasts around 40 ms which is equivalent to 1–2 cycles of slow gamma oscillations. Such oscillations, if properly transmitted from hippocampus over the cortex, can therefore coordinate information transfer. The patterns of slow gamma transmission between and within hippocampal and cortical structures (which we will otherwise call interaction or connectivity patterns) are illustrated in Figs. 2, 3, 4 and 5. The changes in the connections after vs before learning for WT and TG mice are illustrated in Figs. 6 and 7.

It may be useful to clarify here some terms we use in the interpretation of the figures. Synchronization is a phenomenon appearing when two different sites act together in time. For oscillating systems synchronization is occurrence of common frequencies coherent in phase. Coherent in phase means that phases of oscillations in two sites are not random, but there is a stable phase difference between them.Transmission is activity flow from one structure to another. In the context of this work its measure is dDTF. Coupling is a measure of the strengths of interaction between structures. In our case its estimator is dDTF integrated in certain frequency range. dDTF identifies reciprocal connections, it makes possible to differentiate the direction of transmission and coupling from structures: A → B and B → A. The transmission: A → B indicate signals in A proceeding signals in B with some time lag.

In Figs. 2, 3, 4 and 5 the strengths of coupling are illustrated. Coupling for each animal/connection is calculated as an integral of the dDTF function over slow gamma frequency range (20–50 Hz). dDTF is a unit-less function, which takes values from 1 to 0. In practice dDTF values are rather small fractions and only their relations are of importance, therefore dDTF values were multiplied by a number 10^5^ few orders of magnitude increasing their values to avoid handling of small numbers.

### Interaction patterns expressed during SWS/no-SWR before learning

The patterns of slow gamma transmission expressed before learning in the WT mice are illustrated in the Fig. 2A. For clarity of the figure, paths of transmission are shown separately in the following order: from CA1 to the cortex (left), in between the cortices (middle) and from the cortex to CA1 as well as between CA1 ipsi- and contralateral (right).

As illustrated, the main slow gamma output from hippocampus goes to PCC and ACC (see yellow and blue rectangles, Fig. 2A, left). PCC and ACC are thereafter involved in two independent loops of reciprocal interaction, namely with RSC and PFC, respectively (Fig. 2A, middle). Such loops suggest synchronization or nearly synchronization of oscillations between PFC and ACC, as well as between PCC and RSC. However, finally, the activity is transmitted back only from PCC to both CA1 (Fig. 2A, right). This closes the loop of reverberating activity between hippocampus and cortex which contain PCC, RSC and both CA1 that are both sources and receivers of slow gamma activity in this loop (see arrows and rectangles in between pink squares, Fig. 2A. The interaction between hippocampus and cortex is therefore found to be bidirectional. Importantly, transmission of slow gamma oscillations occurs also between the two CA1 (see connections between CA1, Fig. 2A, right). It is worth noticing that this interaction is not transmitted via any sites recorded in this study, since the dDTF method eliminates possibility of indirect interaction between structures considered in the analysis.

Similarly to WT mice, in TG group the main transmission of slow gamma signals from hippocampus targets PCC (see blue and yellow rectangles, Fig. 3A, left). However, the other cortical site which is involved in this transmission is different, i.e. signals reach RSC (see blue rectangle, Fig. 3A, left) and not ACC, as in WT animals (compare Figs. 2A and 3A, left). PCC and RSC are now involved in reverberating loop of cortico–cortical activity which is send back to CA1 ipsilateral (Fig. 3A, middle and right, respectively). Notice the other loop between ACC and PFC which is not involved in hippocampal–cortical–hippocampal interaction since there are no transmission from PCC or RSC to any of these cortices (see lack of arrows, Fig. 3A, middle). Among cortico–cortical interactions the strongest connections is established between ACC and PFC, as in WT group (see yellow rectangles in Figs. 2A and 3A, middle). Stronger reciprocal interaction than in WT mice is expressed between both CA1 (c.f. Figs. 2A and 3A, right).

In summary, in both groups, in the absence of SWRs, the main slow gamma transmission from hippocampus is reaching cortex through PCC. Thereafter the activity is synchronized with other cortices’ activity via cortico–cortical interaction and sent back to hippocampus through connections between PCC (in both groups) and ACC (in TG) with CA1. The strongest cortico–cortical coupling is established between ACC and PFC. Reciprocal interaction between both hippocampal sites is present.

### Effect of learning on the transmission patterns during SWS/no-SWR

In WT group no alteration of the connectivity patterns was expressed after learning (c.f. Fig. 2A,B). In TG mice the effect of learning was very modest and consisted in expressing of a new connection between cortex and hippocampus, namely between ACC and CA1 ipsilateral (c.f. Fig. 3A,B).

### Interaction patterns expressed during SWS/SWR

As illustrated in Fig. 4A, during SWRs episodes, transmission from hippocampus to cortex in WT mice was more robust than during SWS intervals not containing SWRs. Indeed, beside the pathway from CA1 to ACC and PCC which was present in the latter, slow gamma signals are transmitted now also to RSC and PFC (Fig. 4A, left). Moreover, due to new reciprocal connections established between ACC and PCC, all cortical sites became active elements of cortico–cortical transmission constituting several loops of reverberating activity (see connectivity between pink rectangles, Fig. 4A, middle). The transmission of slow gamma oscillations from cortex to hippocampus also increased during the SWRs episodes. The signals are now transmitted not only from PCC but also from ACC to both hippocampal sites, although the strength of interaction is similar (c.f. Figs. 2A and 4A, right). Finally, transmission from the CA1 ipsilateral to CA1 contralateral increased compared to the pattern expressed in absence of SWRs (c.f. Figs. 2A and 4A, right).

Also in TG animals, transmission pathway from hippocampus to cortex strengthened during occurrence of SWRs in term of increasing the number of pathways (8 instead of 3).

Cortico–cortical interaction involved larger number of pathways than during absence of SWRs (i.e., seven vs. six pathways, c.f. Figs. 3A and 5A, middle). Namely, new pathway was established between PCC and ACC so that slow gamma activity is now synchronized among all recorded cortices (c.f. connections between pink squares). Transmission of signals from cortex to hippocampus was quite robust compared to the pattern expressed in absence of SWRs and involves all cortical sizes, each of them targeting both CA1 (c.f. Figs. 3A and 5A, right). Finally, interaction between CA1 was reciprocal of similar strength than during episodes without SWRs occurrence.

In summary, in WT and TG animals the transmission of hippocampal slow gamma oscillations to cortex involves more cortical targets during SWRs than in their absence. In both groups intracortical connectivity increases by involvement of the loop between ACC and PFC. The transmission from cortex to hippocampus still takes place from PCC and ACC in WT mice but is much more robust in TG animals where it involves all possible pathways between the cortex and CA1. Interaction between two hippocampal sites is still reciprocal in both groups.

### Effect of learning on the transmission patterns during SWS/SWR

The post-learning alteration of the connectivity patterns was much more robust in TG than in WT animals. Indeed, whereas in WT mice it consisted only in disappearing of a single connections between ACC and CA1 (c.f. Fig. 4A,B, right). In TG group all intra-cortical pathways became activated, that is the number of active pathways increased from 5 to 12 (c.f. Fig. 5A,B, middle). Beside the strengthening of intracortical connectivity in this group, transmission substantially increased in the pathways from CA1 to cortex and from the cortex to CA1 (c.f. Fig. 5A,B, left and right, respectively). Moreover, and in contrast to WT group, the interaction between two CA1 sites increased in TG animals after visiting the maze (c.f. Fig. 5A,B, right). Thus, counterintuitively, the effect of learning was in practice visible only in TG animals, in which all the pathways allowing bidirectional transmission of slow gamma oscillations between CA1 and recorded cortices became more activated.

The effects of learning in terms of the ratios of the coupling strength expressed after vs before learning, for WT and TG mice are illustrated in Figs. 6 and 7, respectively. They show the same tendencies as these visible in Figs. 2, 3, 4 and 5. For WT mice (Fig. 6) the changes of coupling ratios were quite small, except increase of the coupling from RSC to CA1 ipsi during SWS/SWR and from RSC to PCC during SWS/no-SWR). For TG mice (Fig. 7) during SWS/SWR episodes the remarkable increase of the coupling from CA1 to cortical sites and between cortical structures was expressed after learning.

### Comparison of the connectivity strength: before and after learning, with and without SWRs

**Figure 8:**
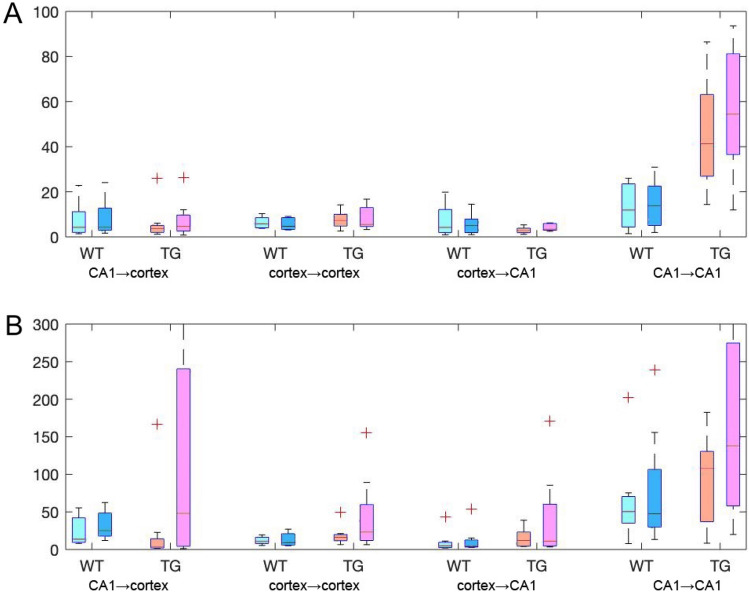
The total strength of interactions expressed between brain structures during episodes not containing SWRs (**A**) and coincident with SWRs (**B**). Shown are (from the left to the right): coupling from CA1 to cortex, between cortices, from cortices to CA1, and between two CA1. On each box, the central mark indicates the median, and the bottom and top edges of the box indicate the 25th and 75th percentiles, respectively. The whiskers extend to the most extreme data points not considered outliers, and the outliers are plotted individually using the ' + ' symbol. Boxes relating to WT mice—before learning light blue, after learning dark blue; boxes relating to TG mice—before learning orange, after learning magenta. Please note the different vertical scales of the upper and lower picture.

Whereas schemes of interaction patterns was detailly described above, information about the strength of connections is still lacking since only a range of the strength values was presented in Figs. 2, 3, 4, 5, 6 and 7. We therefore illustrate in Fig. 8 the total strength of interactions expressed sequentially: between both CA1 and cortex, within cortices, from cortices to CA1 and between two CA1 during SWS/no-SWR and SWS/SWR (Fig. 8A,B, respectively). We present the strengths of coupling by means of boxplots, separately for animal groups and conditions, before or after visit in the maze. The middle line in each boxplot represents the median; the notches are the 95% confidence interval for the median.

There are several conclusions which come out from the inspection of Fig. 8. First, the coupling between considered structures during SWS episodes coincident with SWRs is stronger than during episodes without SWRs, c.f. Fig. 8A,B (please note that the scale in B is 3 times bigger than in A). Indeed the differences in connectivity strength found by means of pair rank Wilcoxon test are significant for majority of connectivity types and conditions (Table 1).

**Table 1:** Comparison of connectivity strength expressed during SWS/no-SWR and SWS/SWR.

	CA1 → cortex	Cortex → cortex	Cortex → CA1	CA1 → CA1
**WT before learning**				
SWS/no-SWR versus SWS/SWR	*p* = 0.0078	*p* = 0.0156	*p* = 0.7422	*p* = 0.0078
**WT after learning**				
SWS/no-SWR versus SWS/SWR	*p* = 0.0078	*p* = 0.0078	*p* = 0.1953	*p* = 0.0078
**TG before learning**				
SWS/no-SWR versus SWS/SWR	*p* = 0.7422	*p* = 0.0078	*p* = 0.0078	*p* = 0.0781
**TG after learning**				
SWS/no-SWR versus SWS/SWR	*p* = 0.0391	*p* = 0.0078	*p* = 0.0156	*p* = 0.0234

Second, the strength of interaction expressed in TG mice was, in general, higher than in WT mice, which is noticeable especially during SWS/SWR episodes (c.f. blue and dark blue (WT) and orange and magenta bars (TG)) Fig. 8B). Finally, most outstandingly, the post-learning alteration of the connectivity strength was found only in TG animals. Indeed, in this group the total transmission of slow gamma oscillations during SWRs from CA1 to cortex increased significantly after learning session (paired rank Wilcoxon test, *p* = 0.02, *N* = 8) as well as did intrahippocampal transmission (paired rank Wilcoxon test, *p* = 0.02, *N* = 8) (Fig. 8B).

## Discussion

In the present study transmission of slow gamma (20–50 Hz) oscillations between hippocampal and cortical circuits during slow wave sleep was analyzed in epochs in which SWRs were generated in the hippocampus and during SWRs’ absence. Using dDTF method, a connectivity measure based on Granger causality, we described main features of the connectivity patterns and how they are affected by encoding new spatial information in 8 to 9-month old WT and TG mice.

The patterns of slow gamma transmission share some features in WT and TG mice independently on SWRs occurrence or experimental situation (i.e. before or after learning). In the two groups propagation of slow gamma oscillations was always bi-directional, forming multiple loops of interaction which involved both CA1 and some of recorded cortical sites. In the absence of SWRs the main transmission from the hippocampus to cortex was directed to PCC, ACC was included in WT group and RSC in TG group as a second cortical target. This is different from^20^ where there was no gamma propagation between ACC and CA1 in WT animals. This difference may be due to time intervals that were chosen in both studies. Indeed, whereas in the present paper all slow wave sleep intervals not coincident with SWRs were taken into account, in the previous paper^20^ only 300 ms intervals before and after SWRs were analyzed. Moreover, it may be due to the different gender of experimental animals (here—male, in^20^—female).

The output from the cortex to hippocampus was established via PCC in WT and via PCC and RSC in TG mice, similarly to what was found in the previous study. During SWRs occurrence the number and strength of hippocampal–cortical and intra-cortical pathways increased in both groups whereas the connectivity between cortex and hippocampus increased only in TG animals;

The effect of learning was expressed only during SWRs. The main difference between the groups consisted in an outstanding strengthening of the slow gamma transmission in the TG group compared to WT mice. Indeed; the propagation from hippocampus to cortex as well as between both CA1 increased significantly in TG mice whereas no significant alteration was found in WT animals.

Networks involved in memory formation are not yet perfectly identified due to the number of structures involved, therefore neither understanding of anatomical connectivity (anatomical links between brain structures) nor understanding of functional connectivity (statistical dependence between activity of brain structures assessed usually by means of correlation or coherence) is sufficient to draw the scheme of the information flow during this process. Herein applying the method based on Granger causality principle we determined the effective connectivity, which shows the causal coupling and direction of propagating activity.

By contrast to a large body of computational models of memory formation which assume the one-way interaction during sleep, from the hippocampus to cortex^28,29^ our results give evidence of multiple bidirectional interactions mediated by slow gamma oscillations, involving both CA1 and different cortices. Among cortices recorded in our study RSC was recently reported as the structure leading the hippocampal gamma events and suggested to initiate hippocampal replay^7^.

Whereas the study^7^ describe bidirectional pseudo-causality between the occurrence of Up- and Down transits in RSC and hippocampal slow gamma, our results show a strong bidirectional coupling between slow gamma oscillations in CA1 and RSC, the coupling that is either direct (see Figs. 3 and 5) or indirect via reciprocal interaction between PCC and RSC (see Figs. 2 and 4). The idea of bidirectional information flow between hippocampus and cortex mediated by oscillations is not new as it was already evidenced that PFC and hippocampus become coupled by theta oscillations during memory processing^7^. Herein we show another frequency band (slow gamma) that can be involved in coordination of bidirectional information transfer between hippocampus and RSC, in accordance with suggestions of^7^.

We may suppose that the CA1 connection to RSC observed by^7^ may be effected through PCC. Although both these structures show dense connections to the hippocampal formation^30^ and projections of hippocampus to RSC exist^31^, we do not know whether the link between slow gamma in CA1 and PCC or RSC is realized solely through these pathways or also via other brain structures, not recorded in this study. However, a strong coupling between RSC and PCC revealed by our analysis may have been expected, since they these structures are anatomically close.

The direct bilateral interaction between PCC and CA1 expressed in both groups and experimental situations support the hypothesis that the cingulate cortex provides a bridge linking neocortical areas with the hippocampal memory system^32^, with spatial information reaching the hippocampal system via the PCC.

The other cortices involved in the bi-directional cortical–hippocampal transmission of slow gamma oscillations, although only during ripples episodes, were ACC (directly in TG and WT) and PFC (directly in TG or via interaction with ACC in WT). The projections of hippocampus to ACC were mentioned by (Chen & Etkin, 2013)^31^, whereas projections in the opposite direction from ACC to CA1 and CA3 fields were reported by (Zemla & Basu, 2017)^33^. PFC and hippocampus are strongly connected by direct and indirect pathways. Indeed, monosynaptic projections from the ventral hippocampus as well as two multi-synaptic pathways via the thalamus and via the perirhinal and lateral entorhinal cortex^34^ connect these two regions. Moreover, a discrete monosynaptic projection from the PFC to dorsal CA1 in mice was recently reported by Malik et al. (2022)^35^ whereas involvement of both these structures in spatial memory consolidation in mice has been documented by Tuscher et al. (2018)^36^.

Interesting phenomenon is the reciprocal coupling between left and right hippocampus indicating a synchronization of slow gamma oscillations, that was expressed in both groups and experimental situations (see Figs. 2, 3, 4, 5, 6 and 7). The robustness of this phenomenon contrasts with the scarce information about anatomical connections between left and right CA1. According to (Guan et al. 2021)^37^ the integration of activity between both CA1 is mediated by the bilateral projections from CA3. Bonds between hippocampal structures has been suggested also using electrophysiological approach showing that ripples developed in both hemispheres virtually simultaneously, however there was no cycle-by-cycle synchrony between them^15,38^. Interestingly, slow gamma synchronization was proposed as a putative mechanism of coordination, both within and across hemispheres, as the sequences of spikes from CA3 and CA1 generated during SWRs in the curse of memory replay^15^. It is therefore supporting this hypothesis that we found the strengthening of the coupling between left and right CA1 for slow gamma coincident with SWRs occurrence.

Counterintuitively, the links between both CA1 were stronger in TG than in WT mice. Moreover, after learning session the interaction between brain structures studied was differently expressed in both groups: whereas the WT mice did not show any changes, the TG group displayed strengthening of slow gamma transmission from CA1 to the cortex and between both CA1 (see Fig. 8). In a previous study it has been shown that 9 month old TG mice were able to consolidate spatial memory despite a pronounce deficit in working memory and advanced neurodegenerative stage of the brain^20^. We may speculate that increased activity exchange between hippocampal structures and from the hippocampus to cortex observed after learning in TG mice serves more efficient transmission of information from impaired CA1 with putatively weakened anatomical bonds.

The structures of the archi- and neocortex resulting from a long evolution of the vertebrate brain have different physiological functions. Slow gamma oscillations may have, in accordance, different timing and functions. It has been shown that in hippocampus slow gamma synchronization is supporting memory formation^15^. We therefore wanted to test whether these hippocampal oscillations are a part of a bigger oscillatory network, involving other brain structures. Using dDTF method we show causal relationships between slow gamma signals recorded in different brain areas involved in memory processing including hippocampus, and we show how they are transmitted during memory task. However, we cannot exclude that the functions of these oscillations are different from memory formation.

Majority of previous studies approaching the connectivity problem in memory addressed fragmentary information about the bonds between the structures involved in memory processes. Herein the evaluation of the relevant signals (slow gamma) in the framework of one model (dDTF) made possible to determine comprehensive scheme of interactions between specific cortical and hippocampal structures.

## Materials and methods

### Study approval

Experiments were approved by CEEA50 Ethical Committee of the University of Bordeaux (authorization no 21,377) and complied with official European guidelines for the care and use of laboratory animals directive. The study was conducted and reported in accordance with ARRIVE guidelines.

#### Animals and surgery

 The data presented in this study were collected from 9 APP/PS1 mice and 8 their Wild Type (WT) littermates age of 8–9 months. These mice were housed in Bordeaux University animal facility (A33063099) under standard conditions (i.e. one animal per cage, temperature (22 ± 1 °C), humidity (50 ± 10%) 12 h light/dark cycle (lights on at 0700), ad libitum access to food and water prior the experimental procedure). Such mice have been used previously by our group^20,27^. All mice were heterozygous for each transgene. The genotypes were confirmed by polymerase chain reaction of tail biopsy. After two weeks of habituation in the experimental animal facility mice underwent stereotaxic surgery under deep isoflurane anesthesia. Microelectrodes, consisting of insulated tungsten wire (diameter 35 μm, California Fine Wires), were implanted using stereotaxic coordinates into: the Prefrontal Cortex (PFC) (AP: + 2.0 mm, L: − 0.36 mm, V: − 1.6 mm), Anterior Cingular Cortex (ACC) (AP: + 0.98 mm, L: − 0.32 mm, V: − 1.48 mm), Posterior Cingular Cortex (PCC) (AP: − 2.0 mm, L: − 0.3 mm, V: − 0.8 mm), Retrosplenial Cortex (RSC) (AP: − 3.0 mm, L: − 0.5 mm, V: − 0.8 mm) and CA1 region of left and right hippocampus (AP: + 2.0 mm, L:  ±  1.5 mm (left or right hemisphere), V: − 1.05 mm ). Reference and ground electrodes were implanted into the cerebellum. The electromyogram (EMG) electrode was inserted into the neck muscles. All electrodes were joined to a 16-pin connector attached to the skull with dental acrylic cement. After surgery animals were housed individually and had 3–4 weeks of recovery before the beginning of recordings and behavioral sessions.

### Experimental procedure

Three to four days before starting behavioral training, mice were put on a partial food restriction diet so that their individual body weights were progressively reduced to 88–85% of the ad libitum weights for running the first session of habituation to the radial-maze. Then the animals were maintained at around 90% of their free feeding weight for one day training by adjusting available food quantity to each mouse.

Access to water remained free. All procedures took place during the light cycle. The animals were exposed to the spatial memory experience for only one day in an elevated eight-arm radial maze (IMETRONIC (Pessac, France)). This maze consisted of a central platform (30 cm in diameter) from which radiated eight identical arms (50 cm long and 11 cm wide). Each arm entrance had an automatic sliding door which was remotely controlled via software by the experimenter located in adjacent room. The rewards delivered at the distal end of the arms were small pellets of pasta. Some distal cues were fixed on the walls of the experimental room. Mice were habituated with the maze and its environment during two habituation sessions (1 day). During the experiment day, first the mice stayed in their home cage with the connector plugged to the recording system during 1–3 h to obtain at least 30 min of sleep. Then, they were unplugged and put into the maze where 3 arms were baited (two adjacent arms and the third one separated by a non-baited arm) (see^20^). Each animal performed six trials. The trial was ended when all the rewards were eaten. Finally, the animals were put back to their home cage where they stayed again 1–3 h connected to the recording system. Configuration of baited arms was randomly assigned to individual.

### Behavioral parameters

Total memory errors for each trial were defined as all visits to any arm of the maze that was not baited and repeated visits to arms that were previously baited in the ongoing trial. Reference memory errors were defined as the number of non-baited arms visited during trial. Working memory errors were defined as the number of revisits to baited arm during trial.

#### Data acquisition

During experiment, the mouse head connector was linked to amplifiers by a soft cable allowing free motions of the animal. Video camera was used to monitor behavior in the home cage. Electrophysiological signals (LFP and EMG) were acquired at 40 kHz on 128-channel Plexon system and stored on a PC for off-line analysis.

### Data processing

Signals were filtered by means of Matlab ‘cheby2’ filter using ‘filtfilt’ function in order to prevent any filter-introduced phase distortions that could undermine the dDTF analysis. Behavioral states of wakefulness, REM sleep and slow wave sleep (SWS) were scored visually by an experienced experimenter using LFP signals, EMG and video recordings. SWS was defined to be a period of high-amplitude activity in the delta band (0.5–3 Hz), low theta activity (4–10 Hz) accompanied by behavioral immobility, as assessed using video recordings, with an absence or weak tonic muscle activity. The ripples were detected in CA1.

Wideband signals were downsampled to 500 Hz using the Matlab procedure ‘decimate’. Signals taken from the CA1 channel, filtered in the 100–250 Hz frequency band were used to detect episodes of SWRs. They were found automatically by thresholding the absolute value of the analytic form of the signals, obtained using Hilbert transform, The signal envelopes found in this way were then z-scored, and segments in which they crossed 2 standard deviations (SD), and reached 5 SD of reference signal values were considered to be ripple events. Events separated by less than 20 ms were merged and events longer than 100 ms were discarded.

**Figure 9:**
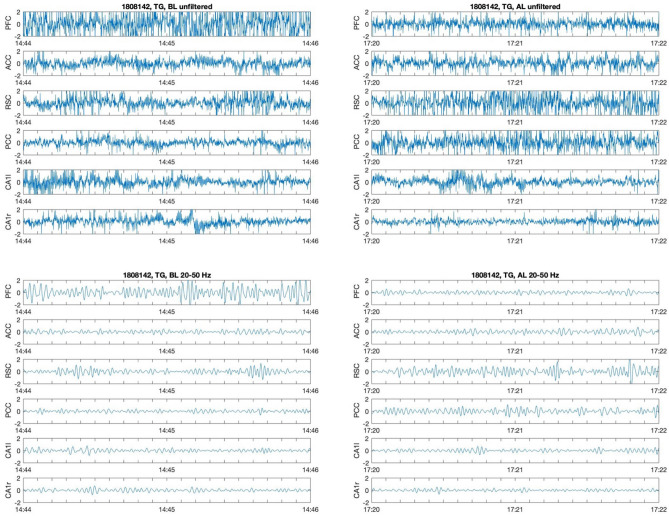
Examples of recorded LFP signals for TG mouse before (left column) and after learning (right column). Two-seconds long epochs selected from SWS recordings are shown. Upper pictures—unfiltered signals, sampled at 500 Hz. Lower pictures—the same signals filtered in slow gamma range (20–50 Hz). Horizontal scale shows time from the beginning of the recording in min:s format.

LFP signals sampled at 500 Hz frequency and the same signals filtered in 20–50 Hz range are shown in Fig. 9.

Two kinds of time series were selected: epochs during which the ripples in CA1 occurred called SWS/SWR and epochs during which they were not identified—SWS/no-SWR. They were processed separately by means of dDTF for WT and TG mice before and after learning.

### Determination of directed coupling between signals

Directed Transfer Function detects causal coupling between multichannel time series, since it is an extension to multichannel case of the Granger causality principle, which was originally introduced for two time series^39^. Granger principle states that for two time series, if the variance of the prediction error for the second time series is reduced by including past measurements from the first time series in the linear regression model, then the first time series can be said to cause the second time series^39^. In case of DTF the formalism was extended to the arbitrary number of time series by fitting to the signals a Multivariate Autoregressive Model (MVAR)^40^. We can express the MVAR model in the form

1 $${\mathbf{X}}(t) = \sum\limits_{m = 1}^{p} {{\mathbf{A}}(m){\mathbf{X}}(t - m) + {\mathbf{E}}(t)}$$

where **X**(*t*) = [*X*_1_(*t*), *X*_2_(*t*),… *X*_*k*_(*t*)]^T^ is the signal vector, **E**(*t*) ) = [*E*_1_(*t*), *E*_2_(*t*),… *E*_*k*_(*t*)]^T^ is the white noise vector (prediction errors), both of size *k* (number of channels), **A**(*m*) are the model coefficients matrices, *p* is the model order.

The MVAR model assumes that **X**(*t*)—a sample of signal at a time *t*—can be expressed as a sum of *p* previous values of signals from the **X**(*t*) vector, weighted by the model coefficients **A**(*m*) plus a prediction error **E**(*t*). The MVAR coefficients are estimated using correlation matrix computed between all considered channels.

In order to calculate MVAR coefficients we used ensemble averaging. The estimation of MVAR coefficients is based on computation of the correlation matrix *R*_*ij*_ of *k* signals *X*_*i*_ from multivariate set. First, for each animal we calculated the correlation matrix between channels for each epoch of *N*_*S*_ points separately. Then we averaged correlation matrices over *N*_*T*_ epochs. The resulting correlation matrix is defined by the equation:

2 $$\tilde{R}_{ij} (s) = \frac{1}{{N_{T} }}\sum\limits_{r = 1}^{{N_{T} }} {R_{ij}^{(r)} (s) = \frac{1}{{N_{T} }}\sum\limits_{r = 1}^{{N_{T} }} {\frac{1}{{N_{S} }}\sum\limits_{t = 1}^{{N_{S} }} {X_{i}^{(r)} (t)X_{j}^{(r)} (t + s)} } }$$

The averaging concerns correlation matrices; the data are not averaged in the process. From *R*_*ij*_ model coefficients were computed by means of known procedures^41^.

From model coefficients the transfer matrix of the model may be found in the form:

3 $$H(f) = \left( {\sum\limits_{m = 0}^{p} {A(m)\exp ( - 2\pi imf\Delta t)} } \right)^{ - 1}$$

where *m* is the number of coefficients, *f* – frequency.

DTF_*ij*_( *f* ) defined by means of **H**( *f* ):

4 $${\text{DTF}}_{ij} (f) = \frac{{\left| {H_{ij} (f)} \right|^{2} }}{{\sum\nolimits_{m = 1}^{k} {\left| {H_{im} (f)} \right|^{2} } }}.$$

describes causal influence of channel *j* on channel *i* at frequency *f* normalized in respect of inflows to the destination channel *i*.

DTF expressed by formula (4) shows not only direct, but also indirect (cascade) flows. To determine only direct flows, direct DTF (dDTF) was introduced^42^.

5 $${\text{dDTF}}(f) = {\text{ffDTF}}(f)\cdot\;P(f)$$

ffDTF is the version of DTF, where the dependence of denominator in Eq. (4) on frequency was abandoned. Partial coherence *P*( *f* ) has properties similar to ordinary coherence, however it is nonzero only when the given relation between channels is direct.

The MVAR model was fitted for each animal separately, commonly for SWS/no-SWR and SWS/SWR epochs, before and after learning, using ensemble averaging for coefficients estimation. Then dDTF functions were determined. In this way for each animal and each condition we obtained the matrix of dimension 6 × 6 of dDTF( *f* ) functions showing transmissions between all channels. In order to find the coupling strengths *C*_*ij*_ between considered channels, we integrated the dDTF( *f* )s in the band of interest, namely in the slow gamma (20–50 Hz) range. Then the *C*_*ij*_ values were averaged over animals.

For statistical analysis the distributions of single transmissions were used. We applied Wilcoxon signed rank tests to determine significances of differences between transmissions before and after learning or transmission during SWS/no-SWR and SWS/SWR epochs.

To get global strengths of transmissions we sorted structures in the groups: CA1 and cortex, by averaging couplings between channels belonging to each group. Then we inspected transmissions between: CA1 → cortex, cortex → cortex, cortex → CA1 and CA1 → CA1 in both directions.

## Data Availability

The datasets used and analyzed during the current study are available from the corresponding author on reasonable request.
